# Revealing the novel autophagy-related genes for ligamentum flavum hypertrophy in patients and mice model

**DOI:** 10.3389/fimmu.2022.973799

**Published:** 2022-10-05

**Authors:** Peng Li, Cheng-shuo Fei, Yan-lin Chen, Ze-sen Chen, Zhong-ming Lai, Rui-qian Tan, Yong-peng Yu, Xin Xiang, Jia-le Dong, Jun-xiong Zhang, Liang Wang, Zhong-min Zhang

**Affiliations:** ^1^ Division of Spine Surgery, Department of Orthopedics, Nanfang Hospital, Southern Medical University, Guangzhou, China; ^2^ Department of Orthopedics, The Third Affiliated Hospital, Southern Medical University, Academy of Orthopedics, Guangzhou, China

**Keywords:** ligamentum flavum hypertrophy, degenerative lumbar spinal stenosis, autophagy, fibrosis, bioinformatics analysis, bipedal standing mouse

## Abstract

**Background:**

Fibrosis is a core pathological factor of ligamentum flavum hypertrophy (LFH) resulting in degenerative lumbar spinal stenosis. Autophagy plays a vital role in multi-organ fibrosis. However, autophagy has not been reported to be involved in the pathogenesis of LFH.

**Methods:**

The LFH microarray data set GSE113212, derived from Gene Expression Omnibus, was analyzed to obtain differentially expressed genes (DEGs). Potential autophagy-related genes (ARGs) were obtained with the human autophagy regulator database. Functional analyses including Gene Ontology (GO), Kyoto Encyclopedia of Genes and Genomes (KEGG) enrichment, Gene Set Enrichment Analysis (GSEA), and Gene Set Variation Analysis (GSVA) were conducted to elucidate the underlying biological pathways of autophagy regulating LFH. Protein-protein interaction (PPI) network analyses was used to obtain hub ARGs. Using transmission electron microscopy, quantitative RT-PCR, Western blotting, and immunohistochemistry, we identified six hub ARGs in clinical specimens and bipedal standing (BS) mouse model.

**Results:**

A total of 70 potential differentially expressed ARGs were screened, including 50 up-regulated and 20 down-regulated genes. According to GO enrichment and KEGG analyses, differentially expressed ARGs were mainly enriched in autophagy-related enrichment terms and signaling pathways related to autophagy. GSEA and GSVA results revealed the potential mechanisms by demonstrating the signaling pathways and biological processes closely related to LFH. Based on PPI network analysis, 14 hub ARGs were identified. Using transmission electron microscopy, we observed the autophagy process in LF tissues for the first time. Quantitative RT-PCR, Western blotting, and immunohistochemistry results indicated that the mRNA and protein expression levels of FN1, TGFβ1, NGF, and HMOX1 significantly higher both in human and mouse with LFH, while the mRNA and protein expression levels of CAT and SIRT1 were significantly decreased.

**Conclusion:**

Based on bioinformatics analysis and further experimental validation in clinical specimens and the BS mouse model, six potential ARGs including *FN1*, *TGFβ1*, *NGF*, *HMOX1*, *CAT*, and *SIRT1* were found to participate in the fibrosis process of LFH through autophagy and play an essential role in its molecular mechanism. These potential genes may serve as specific therapeutic molecular targets in the treatment of LFH.

## Introduction

Degenerative lumbar spinal stenosis (DLSS) is one of the most commonly diagnosed and treated conditions among the elderly population (1). According to estimates, there are approximately 200,000 cases of DLSS in adult Americans (2). The typical clinical symptoms of DLSS are intermittent neurogenic claudication and buttock and lower extremity pain, which may inflict a tremendous burden on the social health-care system worldwide (2, 3). Ligamentum flavum hypertrophy (LFH) is considered one of the major causes of DLSS (4). The proliferated ligamentum flavum (LF) compresses the nerve root or cauda equina nerve, causing numbness and pain in the lower limbs (5). Although a growing number of studies believe that LFH may be related to various multifactorial processes such as fibrosis, inflammation, and mechanical stress (6–8), its exact mechanism remains poorly understood.

A close relationship exists between LFH and fibrosis. Fibrosis has been identified as the central pathology of LFH (7). Histologically, the normal LF is an elastic structure that is composed of elastic (80%) and collagen (20%) fibers (9). As hypertrophy progresses, the LF shows loss of elastic fibers and an increased number of collagen fibers, suggesting fibrotic changes (10). The formation of LFH is the result an abnormal scar healing process, which is characterized by the excessive deposition of extracellular matrix (ECM) proteins by persistently activated fibroblasts (6).

Autophagy is a highly conserved biological process associated with lysosome-dependent self-renewal (11). Through this mechanism, cytoplasm containing aggregated proteins and abnormal organelles are isolated into autophagosomes, which are then delivered to lysosomes for degradation (12). To date, several modes of autophagy have been documented, including macroautophagy, microautophagy, chaperone mediated autophagy, and noncanonical autophagy (13). Under normal physiological conditions, autophagy is a self-defense mechanism of cells (14). Moderate autophagy helps maintain a stable intracellular environment and cope with an adverse environment. Nevertheless, excessive autophagy under pathological conditions can cause excessive degradation of cellular contents, which leads to a kind of cell death known as ‘autophagic cell death’ (15). Autophagy has been confirmed to play a crucial role in the pathogenesis of multi-organ fibrosis, including cardiac fibrosis (16), pulmonary fibrosis (17), and renal fibrosis (18).

Despite autophagy being associated with fibrosis in different tissues, there is no report that autophagy contributes to the pathogenesis of LFH. In this study, we explored the relationship between autophagy and fibrosis progression in LFH for the first time and identified potential autophagy-related genes (ARGs) through bioinformatics analysis and experimental verification in clinical specimens and a bipedal standing mouse model.

## Materials and methods

### Microarray data and ARGs datasets

The LFH microarray data set GSE113212 was obtained from the National Centre of Biotechnology Information Gene Expression Omnibus database (GEO, https://www.ncbi.nlm.nih.gov/geo/), including four LFH samples derived from elderly individuals and four non-LFH samples from young individuals. The Agilent-039494 SurePrint G3 Human GE v2 8x60K Microarray 039381 (Probe Name version; Agilent Technologies, Inc; Palo Alto, CA, USA) platform was used, and the annotation information of the platform was also downloaded from GEO. A total of 796 genes were obtained from the Human Autophagy Moderator Database (http://hamdb.scbdd.com/).

### Differential expression analysis of ARGs

Quantile normalization of the original data was performed, followed by data processing to determine the differentially expressed genes (DEGs) between the LFH samples and normal controls. The limma R package in Bioconductor 4.1 (https://www.bioconductor.org/pack-ages/release/bioc/html/limma.html) was adopted to conduct the quantile normalization of the raw data and subsequent data processing to identify the DEGs between the LFH samples and the normal controls.

Annotation information from the platform was used to transform probes into gene symbols. Through principal component analysis, the repeatability of the data in GSE113212 was verified. R package ‘limma’ was used to identify the DEGs. To analyze the DEGs between the two groups, *t*-tests were used and the *P*-values were adjusted for the false discovery rate using the Benjamini-Hochberg procedure (19). Only genes with a |log_2_fold change | > 1 and *P*-value < 0.05 were selected. The heatmap, volcano plot, and box plot were generated using ‘heatmap’ and ‘ggplot2’ packages in R software.

### Gene ontology (GO) and Kyoto encyclopedia of genes and genomes (KEGG) pathway enrichment analyses

GO is a bioinformatics tool that annotates genes, gene products, and sequences according to specific terms (20). Three categories are included in the GO analysis: biological process (BP), cellular component (CC), and molecular function (MF). KEGG is a data repository for unraveling advanced biological functions and pathways associated with genomic information (21). ClusterProfiler V3.8 is a bioconductor-dependent R package that automates the process of biological-term classification and the enrichment analysis of gene clusters (22). This study used the clusterProfiler package for enrichment analysis of identified ARGs following GO and KEGG analyses.

### Gene set enrichment analysis (GSEA) and gene set variation analysis (GSVA)

GSEA is a method of interpreting genome-wide expression profiles that focuses on evaluating the distribution trend of genes from a predefined gene set ranked according to their phenotypic correlation (23). The clusterProfiler R package was used for GSEA (22). The GO gene sets database (c5.all.v5.1.symbols.gmt) in the Molecular Signatures Database (MSigDB) (24) was used to identify significantly enriched biological processes between the non-LFH and LFH groups. The gene sets database (c2.all.v5.0.symbols.gmt) obtained from MSigDB were also selected as the reference gene sets to conduct significantly enriched signaling pathway analyses. GO terms and pathways with a false discovery rate < 0.25 and a |normalized enrichment score| > 1 were considered as enriched. The GSEA enrichment plots were generated by the R package ‘enrichplot’.

GSVA is a gene set enrichment method for estimating pathway activity over a sample population in an unsupervised manner (25). The ‘GSVA’ package in R was used for GSVA analysis, which calculated the enrichment score of each sample in each gene set and obtained the enrichment score matrix. the gene set file of BPs (c5.all.v5.1.symbols.gmt) from MSigDB was used to evaluate biological pathways. The heatmap was generated using the ‘heatmap’ package in R software.

### Protein-protein interaction (PPI) analysis and correlation analysis

The STRING database (www.string-db.org) is an online biological database of known and predicted PPIs with the ability to visualize processes and interactions among proteins (26). Based on the PPIs obtained for the identified ARGs, PPI pairs with a combined score over 0.4 for the ARGs were selected from the STRING database. Cytoscape v 3.7.2 (https://cytoscape.org/) was then used to visualize the PPI network and identify the hub ARGs responsible for controlling physiological process. In a PPI network, nodes represent proteins while edges show interactions among these proteins. CytoHubba (http://apps.cytoscape.org/apps/cytohubba/) is an effective app in the Cytoscape plug-in used to accurately identify hub genes by 12 topological analysis methods, including maximal clique centrality (MCC), density of maximum neighborhood component (DMNC), maximum neighborhood component (MNC), and Degree (27). The correlation analysis of differentially expressed ARGs was performed using the R package ‘Corrplot’.

### Clinical specimens information

From July 2021 to January 2022, a total of eight patients with lumbar disc herniation (LDH) and eight patients with DLSS were collected in Nanfang Hospital, Southern Medical University (Guangzhou, China). Human LF specimens in this study were collected according to the protocol (NFEC-2022-175) approved by the Ethics Committee of Nanfang Hospital, Southern Medical University. Written informed consent was obtained from all subjects. All LF specimens from the 16 patients were taken from the dorsal side of the LF at L4/5 during surgery and measured by magnetic resonance imaging (MRI). LF specimens obtained from LDH patients with thickening of the LF (LF thickness ≤ 3.74 mm confirmed by MRI scan) were assigned to the non-LFH group, while pathological LF specimens obtained from DLSS patients with thickening of the LF (LF thickness > 3.74 mm confirmed by an MRI scan) were assigned to the LFH group (28). Patients with spondylolisthesis, ankylosing spondylitis, or spinal tumors were excluded from the study. The LF specimens obtained from 16 patients were each cut in half, and finally 32 LF specimens were obtained. Four of 32 LF specimens were temporarily stored in 2.5% glutaraldehyde (n = 4), 12 of 32 LF specimens were temporarily stored in phosphate buffered saline (PBS) solution (n = 12), and the remainder were stored in liquid nitrogen (n= 16). LF specimens for each experiment used in this study are listed in **Supplementary Table 1**.

### Transmission electron microscopy (TEM) of mitochondrial autophagy structures in the LF specimens

The autophagy structures were observed using a transmission electron microscope (H-7500, Hitachi Technology, Tokyo). LF specimens (non-LFH group:LFH group = 2:2) were obtained and fixed in 2.5% glutaraldehyde working solution. Various autophagy structures, such as phagocytes, autophagosomes, and autolysosomes, were observed at high magnification (×40000) in the non-LFH and LFH specimens.

### Total RNA extraction and quantitative RT-PCR (qRT-PCR)

Both groups of LF specimens (non-LFH group:LFH group = 6:6) were subjected to qRT-PCR. Fat and bone tissue were removed from the epidural space, and LF specimens were rinsed with PBS solution to remove blood. According to standard protocols, the whole specimens were immediately shock frozen in liquid nitrogen, crushed, and dissolved in Trizol solution (Invitrogen, Carlsbad, CA, USA) for total RNA extraction. Total RNA was extracted from quick-frozen LF specimens using Trizol reagent (Invitrogen) according to the manufacturers protocol. cDNA was synthesized using the PrimeScript RT Master Mix (Takara, RR036A, Japan) according to the manufacturer’s instructions. qRT-PCR was performed using the QuantStudio5 (Applied Biosystems, Thermo Fisher Scientific, USA) and SYBR Green Master Mix (Takara, RR820A, Japan). *GAPDH* mRNA was used as the internal normalization control and the 2^−△△Ct^ method was used for relative mRNA calculations. Primer sequences are shown in **Supplementary Table 2**.

### Western blotting

Clinical LF specimens (non-LFH group:LFH group = 2:2) were snap-frozen in liquid nitrogen and stored at −80°C for Western blotting. Total protein from each LF specimen was extracted in RIPA lysis buffer (Santa Cruz) and quantified using the BCA assay (Pierce). After denaturation, proteins specimens were separated using gel electrophoresis on 8%–12% SDS-PAGE, and then were transferred onto PVDF membranes (Roche Applied Science, Indianapolis, IN, USA). Using 5% nonfat dry milk for 2 hours at room temperature, The membranes were blocked in 5% nonfat dry milk for 2 h and then incubated overnight at 4°C with the following primary antibodies: Beclin1 (1:500; AF5128, Affinity), P62 (1:500; AF5384, Affinity), FN1 (1:500; AF5335, Affinity), TGFβ1 (1:500; AF1027, Affinity), NGF (1:500; AF5172, Affinity), HMOX1 (1:500; AF5393, Affinity), CAT (1:500; DF7545, Affinity), SIRT1 (1:500; TU365233, Abmart), and GAPDH (1:5000; AP0063, Bioworld). After, the membranes were incubated with goat anti-rabbit IgG (H+L) HRP secondary antibody (1:5000; RM3002, Rayantibody) for 2 h at room temperature. The proteins bands were detected using an enhanced chemiluminescence kit (KF005, Affinity), and chemiluminescence signals were quantified with Image Lab statistical software (Bio-Rad, Hercules, CA, USA).

### Mouse experiments

All animal experimental protocols were approved by the Animal Ethical Committee of Laboratory Animals of Southern Medical University (NFYY-2021-1021). The 8-week-old male C57BL/6 mice were purchased from the Experimental Animal Center of Southern Medical University (Guangzhou, China). To establish mice LFH models, mice were induced to adopt a bipedal standing posture for 6 hour a day with an interval of 2 hour by taking advantage of the hydrophobia of mice (29). Twelve mice were randomly divided into the control group (n = 6) and the bipedal standing (BS) group (n = 6) for the purpose of validating the mouse LFH model. Within 12 weeks of modeling, each experimental mouse was euthanized, and their intact L5/6 vertebrae was harvested for hematoxylin and eosin (HE) staining, Elastica van Gieson (EVG) staining, and immunohistochemistry (IHC).

### Histological studies and immunohistochemical staining

Human LF specimens (non-LFH group:LFH group = 6:6) and mouse lumbar specimens (control group:BS group = 6:6) were fixed with 4% paraformaldehyde, decalcified in decalcifying liquid, and dehydrated in graded alcohols. All tissues were embedded in paraffin and cut into 4 µm-thick sections. After dewaxing and rehydrating, the sections were stained according to the procedures of HE kits (Sigma-Aldrich, St. Louis, MO, USA) or EVG kits (Service-Bio, Shanghai, China).

Additionally, paraffin sections were dewaxed and rehydrated prior to IHC. For antigen recovery, human tissue sections were microwaved in EDTA buffer (pH= 8) and mouse sections were microwaved in citrate buffer (pH= 6) for a total of 3 min. Endogenous peroxidase activity was blocked with 3% hydrogen peroxide for 15 min in the dark and non-specific binding was blocked for 1 h with ready-to-use goat serum (AR0009, Boster, China). Sections were stained with antibodies against Beclin1 (1:100; AF5128, Affinity), P62 (1:100; AF5384, Affinity), FN1 (1:100; AF5335, Affinity), TGFβ1 (1:100; AF1027, Affinity), NGF (1:100; AF5172, Affinity), HMOX1 (1:100; AF5393, Affinity), CAT (1:100; DF7545, Affinity), SIRT1 (1:100; TU365233, Abmart) overnight at 4°C and then incubated with goat anti-rabbit IgG (H + L) HRP secondary antibodies (BF03008X, Biodragon) for 2 h at room temperature. DAB (Service-Bio, Shanghai, China) was used for color development, and hematoxylin was used as counterstain.

Images of the stained slides were obtained with Olympus BX63 microscope (Olympus, Tokyo, Japan). Using Image J software (NIH, United States), we quantified the LF areas, the ratio of elastic fibers to collagen fibers, and the ratio of positively stained cells in the human and mouse LF sections.

### Statistical analysis

All the statistical analyses were executed by R software (version 4.1.0), SPSS 20.0 software (SPSS Inc., Chicago, IL, USA), and GraphPad Prism 9.0.0 Software (GraphPad Software, La Jolla, CA, USA). The data are presented as mean ± standard error of the mean for all parameters measured. The results were compared by performing Student’s *t*-tests; *P* < 0.05 was considered to indicate statistical significance.

## Results

### Identification of 70 ARGs in human LFH samples compared with control samples

**Figure 1:**
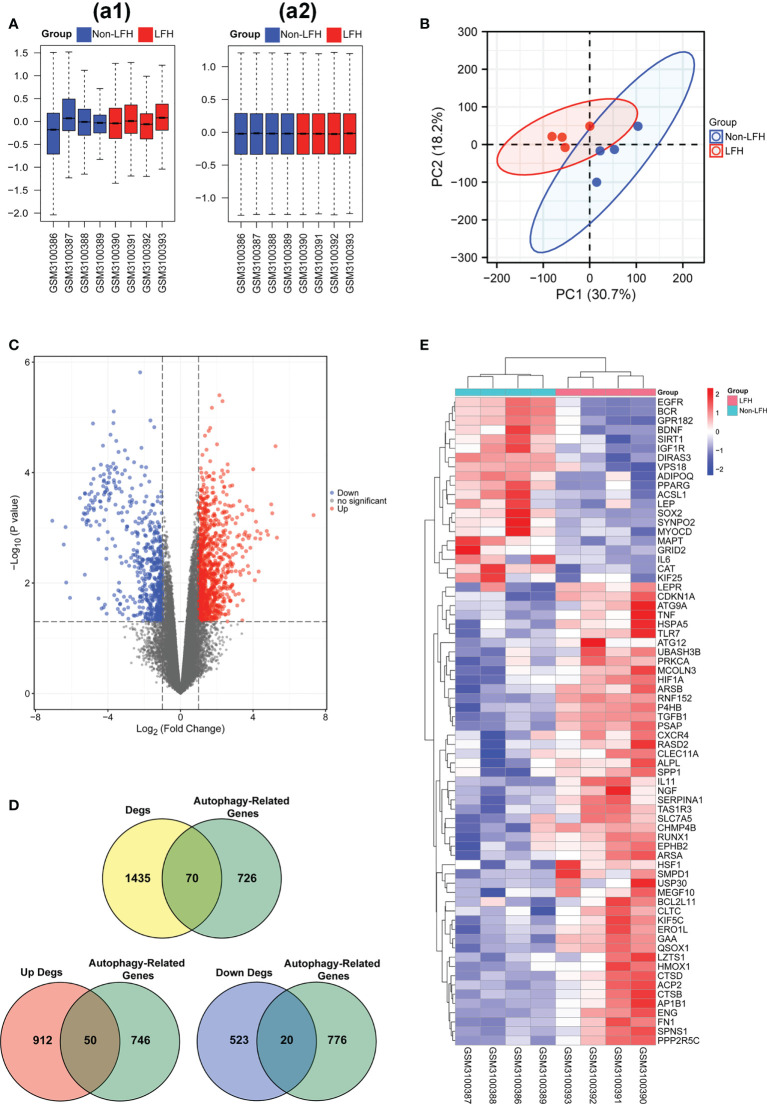
Data preprocessing of microarray data and differentially expressed ARGs in the LFH microarray data set. **(A)**, a1, The boxplot of the LFH microarray data set GSE113212 before sample data standardization. a2, The boxplot of the LFH microarray data set GSE113212 after sample data standardization. **(B)** Principal component analysis of the LFH microarray data set GSE113212. **(C)** Volcano plot of 70 differentially expressed ARGs. Red refers to up-regulated expression. Blue refers to down-regulated expression. Gray indicates no difference in expression. **(D)** Venn diagrams indicating 50 up-regulated and 20 down-regulated genes. Yellow indicates the 1505 differentially expressed genes. Green indicates the 796 ARGs. Blue indicates the 543 differentially up-regulated genes. Red indicated the 962 differentially down-regulated genes. **(E)** Heatmap of the 70 differentially expressed ARGs in the non-LFH and LFH samples. non-LFH = Non-ligamentum flavum hypertrophy; LFH = Ligamentum flavum hypertrophy.

**Figure 2:**
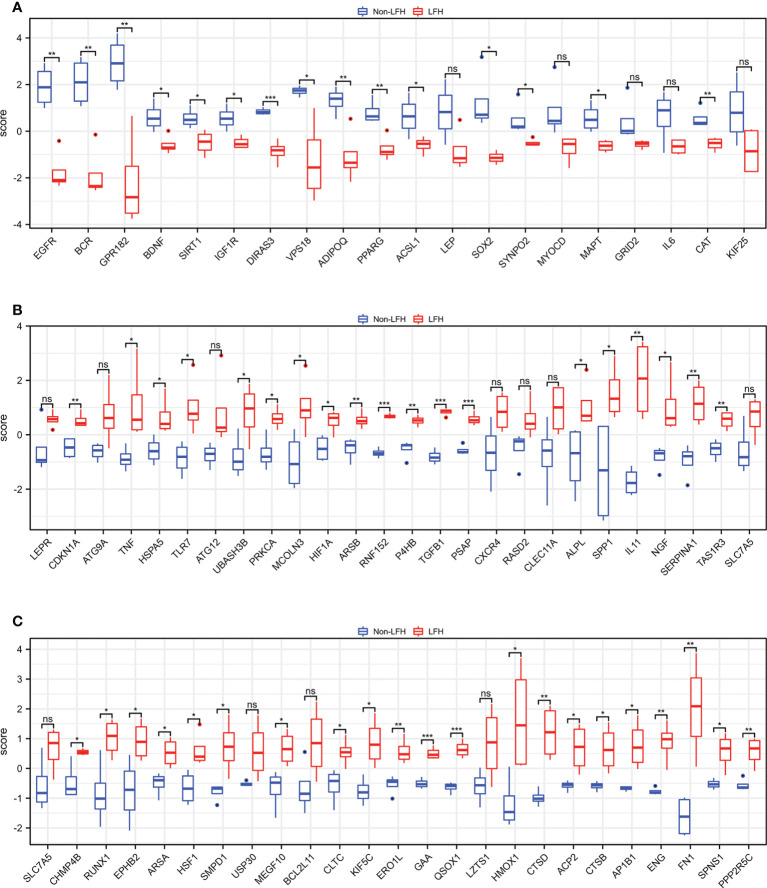
The boxplot of 70 differentially expressed ARGs in the LFH microarray data set. **(A)** The boxplot of 20 down-regulated ARGs between the non-LFH and LFH samples. **(B)**, and **(C)** The boxplot of 50 up-regulated ARGs between the non-LFH and LFH samples. ns, not significant; *P < 0.05, **P < 0.01, ***P < 0.001.

To evaluate the repeatability of data within the group, we normalized the expression data of the LFH microarray data set GSE113212 (**Figure 1A**) and employed principal component analysis. Based on the PCA results (**Figure 1B**), LFH individuals in this dataset differed significantly from non-LFH individuals, indicating the possibility for further analysis. We next analyzed the differential expression of genes and found that 1,505 DEGs were obtained based on a |log_2_fold change (FC)| > 1 and *P*-value < 0.05 as the standard (**Figure 1C**). The intersection of 1505 DEGs and 796 ARGs was used to obtain 70 differentially expressed ARGs, including 50 up-regulated and 20 down-regulated genes (**Figure 1D**) (**Supplementary Table 3**). The 70 differentially expressed ARGs are shown in a heatmap plot (**Figure 1E**). In addition, the box diagram shows the expression patterns of 70 differentially expressed ARGs between non-LFH and LFH samples (**Figures 2A–C**).

### GO and KEGG pathway analyses of the differentially expressed ARGs

**Figure 3:**
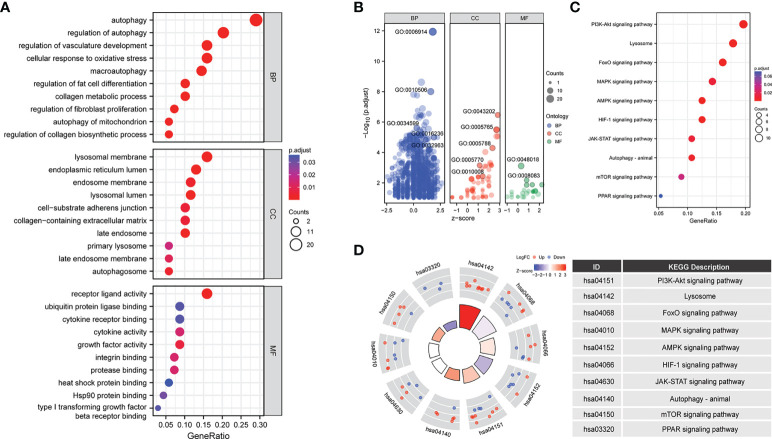
Enrichment analysis of 70 differentially expressed ARGs in the LFH microarray data set. The bubble plot shows the gene Ontology (GO) enrichment analysis of 70 differentially expressed ARGs. **(A, B)** bubble plot of enriched GO terms. **(C)** The bubble plot shows the Kyoto Encyclopedia of Genes and Genomes (KEGG) analysis of 70 differentially expressed ARGs. **(D)** The chord plot shows the KEGG analysis of 70 differentially expressed ARGs. BP, biological process; CC, cellular component; MF, molecular function.

To further analyze the biological functions of the 70 ARGs, we performed GO and KEGG analyses. Functional enrichment analysis showed that in the BP category, the 70 differentially expressed ARGs were mainly enriched in the following GO terms: autophagy, cellular response to oxidative stress, macroautophagy, collagen metabolic process, regulation of fibroblast promotion, and autophagy of mitochondrion. In the CC category, the differentially expressed ARGs were enriched in lysosomal membrane, endosome membrane, lysosomal lumen, collagen containing extracellular matrix, and autophagosome. In the MF category, the genes were enriched in receptor ligand activity, ubiquitin protein ligase binding, cytokine receptor binding, integrin binding, type I transforming growth factor, and beta receptor binding (**Figures 3A, B**) (**Table 1**).

**Table 1:** The Results of GO Enrichment Analysis of ARGs in LFH.

Ontology	Term	Description	Gene Ratio	*P-*value
BP	GO:0006914	autophagy	20/69	7.28e-16
BP	GO:0061919	process utilizing autophagic mechanism	20/69	7.28e-16
BP	GO:0031667	response to nutrient levels	17/69	2.29e-12
BP	GO:0010506	regulation of autophagy	14/69	1.26e-11
CC	GO:0043202	lysosomal lumen	8/69	1.41e-09
CC	GO:0005765	lysosomal membrane	11/69	3.85e-08
CC	GO:0098852	lytic vacuole membrane	11/69	3.96e-08
CC	GO:0005775	vacuolar lumen	8/69	1.51e-07
MF	GO:0048018	receptor ligand activity	11/69	2.39e-06
MF	GO:0008083	growth factor activity	6/69	4.13e-05
MF	GO:0002020	protease binding	5/69	1.42e-04

GO, indicates gene ontology; LFH, Ligamentum flavum hypertrophy; BP, biological process; CC, cellular component; MF, molecular function.

In addition, KEGG biological pathway analysis showed that ARGs were mainly enriched in 10 signaling pathways including PI3K-Akt, FoxO, MAPK, AMPK, HIF-1, JAK-STAT, mTOR, and PPAR signaling pathway (**Figures 3C, D**).

### GSEA and GSVA of differentially expressed genes in LFH

**Figure 4:**
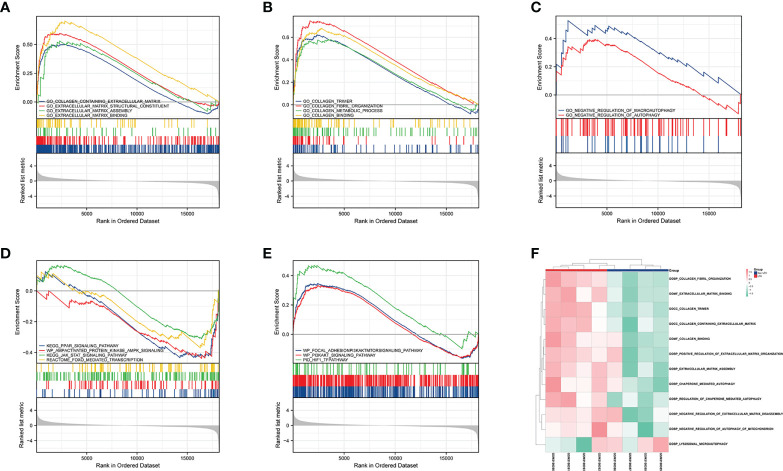
GSEA and GSVA of DEGs in the LFH microarray data set. **(A)** The GSEA enrichment plot indicates that BPs relating to ECM are enriched in the LFH group. **(B)** The GSEA enrichment plot indicates that BPs relating to collagen are enriched in the LFH group. **(C)** The GSEA enrichment plot indicates that BPs relating to negative regulation of autophagy are enriched in the LFH group. **(D)** GSEA reveals four signaling pathways enriched in the non-LFH group. **(E)** GSEA reveals three signaling pathways enriched in the LFH group. **(F)** Heat map shows the abundance of biological processes calculated by GSVA in both the non-LFH and LFH groups. BPs; biological processes; GSEA, Gene set enrichment analysis; GSVA, Gene set variation analysis; ECM, extracellular matrix.

Taking into account the limitations of GO and KEGG enrichment analyses (23), the LFH microarray data set was evaluated by GSEA and GSVA. GSEA allowed us to further analyze the GO terms and KEGG pathways of previous enrichment analyses and clarify their enrichment in different groups. The results of GSEA demonstrated that the non-LFH group was enriched with pathways including FoxO, AMPK, JAK-STAT, and PPAR signaling (**Figure 4D**), while the LFH group was enriched with pathways including HIF-1, PI3K/AKT, and PI3K/AKT/mTOR signaling (**Figure 4E**). The GSEA enrichment plot indicates that the LFH group was enriched with BPs including ECM assembly, ECM binding, collagen binding, and collagen fibril organization (**Figures 4A, B**). In addition, negative regulation of autophagy and negative regulation of macroautophagy were also enriched in the LFH group (**Figure 4C**). A summary of the GSEA results are shown in **Table 2**.

**Table 2:** The Results of GSEA of DEGs in LFH.

ID	NES	FDR	*P-*value
GO_COLLAGEN_CONTAINING_EXTRACELLULAR_MATRIX	2.190389	0.069083	0.001499
GO_EXTRACELLULAR_MATRIX_STRUCTURAL_CONSTITUENT	2.390107	0.069083	0.001572
GO_EXTRACELLULAR_MATRIX_ASSEMBLY	1.678941	0.103554	0.008503
GO_EXTRACELLULAR_MATRIX_BINDING	2.383645	0.069083	0.001642
GO_COLLAGEN_CONTAINING_EXTRACELLULAR_MATRIX	2.190389	0.069083	0.001499
GO_COLLAGEN_FIBRIL_ORGANIZATION	2.489061	0.069083	0.001658
GO_COLLAGEN_METABOLIC_PROCESS	2.163448	0.069083	0.001618
GO_COLLAGEN_BINDING	2.381586	0.069083	0.001678
GO_NEGATIVE_REGULATION_OF_MACROAUTOPHAGY	1.582074	0.131758	0.013769
GO_NEGATIVE_REGULATION_OF_AUTOPHAGY	1.440702	0.185117	0.025848
KEGG_PPAR_SIGNALING_PATHWAY	-1.603715	0.091687	0.015385
WP_AMPACTIVATED_PROTEIN_KINASE_AMPK_SIGNALING	-1.610998	0.090774	0.01519
KEGG_JAK_STAT_SIGNALING_PATHWAY	-1.291394	0.218308	0.060519
REACTOME_FOXO_MEDIATED_TRANSCRIPTION	-1.349606	0.231143	0.065823
WP_FOCAL_ADHESIONPI3KAKTMTORSIGNALING_PATHWAY	1.483075	0.041501	0.004354
WP_PI3KAKT_SIGNALING_PATHWAY	1.428864	0.057698	0.007396
PID_HIF1_TFPATHWAY	1.647005	0.034083	0.003295

GSEA, Gene set enrichment analysis; NES, normalized enrichment score; FDR, false discovery rate.

The results of GSVA showed that lysosomal microautophagy of mitochondrion was enriched in the non-LFH group, whereas the LFH group had a higher abundance of collagen fibril organization, positive regulation of extracellular matrix organization, and negative regulation of autophagy of mitochondrion. Combined with the GSEA analysis results, ECM and collagen-related biological pathways were up-regulated in the LFH group, whereas autophagy-related biological pathways were down-regulated.

### The PPI network analysis and hub ARGs identification

**Figure 5:**
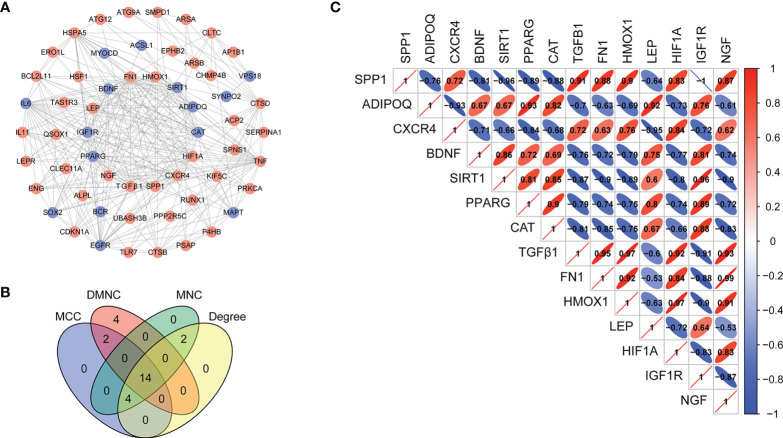
Construction of the PPI network and identification of hub ARGs. **(A)** The PPI between 70 differentially expressed ARGs was constructed by using the STRING database. Genes are represented by nodes, and relationships between genes are represented by edges. Up-regulation of gene expression is represented by the red balls, whereas down-regulation is represented by the blue balls. **(B)** Fourteen hub ARGs were identified *via* a Venn diagram. **(C)** Spearman correlation analysis of the 14 differentially expressed ARGs.

To establish functional relationships among the differentially expressed ARGs and further identify the hub ARGs, we constructed a PPI network using the STRING database (confidence > 0.4) and visualized the network using Cytoscape software. The network consists of 58 nodes and 276 edges, which indicated an interaction between the identified ARGs (**Figure 5A**). The top 20 genes were predicted by MCC, MNC, DMNC, and Degree. The 14 hub ARGs were selected by using Venn diagrams (**Figure 5B**), including *FN1*, *TGFβ1*, *CAT*, *NGF*, *HMOX1*, *SIRT1*, *PPARG*, *IGF1R*, *LEP*, *SPP1,ADIPOQ*, *BDNF*, *CXCR4*, and *HIF1A*. Spearman correlation analysis was then used to analyze the 14 hub differentially expressed ARGs (**Figure 5C**).

### Verification of the differences in autophagy between the non-LFH and LFH groups

**Figure 6:**
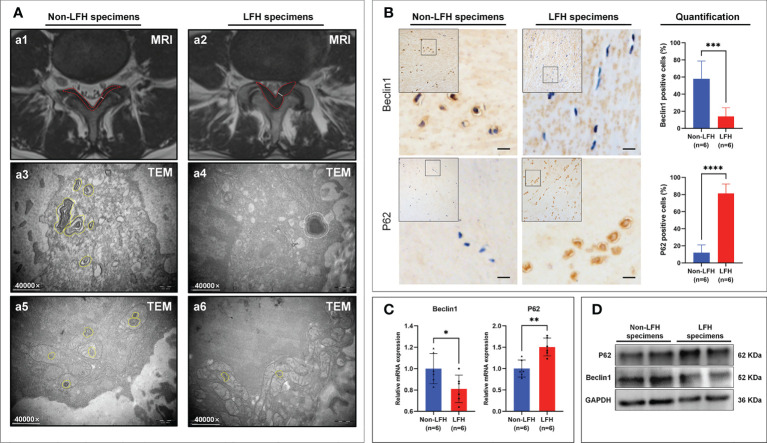
The Differences in Autophagy were validated between the non-LFH and LFH specimens. **(A1, A2)** Measurement of the LF thickness by MRI preoperatively. The white solid line indicates the thickness of the ligamentum flavum at the facet joint level. The red dotted areas represent MRI images of LF. **(A3, A5)** A higher number of autophagosomes was observed in the cytoplasm and mitochondria of non-LFH specimens. The yellow dotted areas represent autophagosomes. **(A4, A6)** Fewer autophagosomes were observed in the cytoplasm and mitochondria of LFH specimens. The yellow dotted areas represent autophagosomes and the white dotted areas represent autolysosomes. **(B)** The autophagy‐related markers Beclin1 and p62 were evaluated by IHC. **(C)** qRT-PCR showed the mRNA levels of *Beclin1* and *p62.* **(D)** Western blotting showed the protein levels of Beclin1 and p62. *P < 0.05, **P < 0.01, ***P < 0.001, ****P < 0.0001.

The thickness of non-LFH tissue was detected by MRI to confirm that the LF thickness was ≤ 3.74 mm (**Figure 6A1**) and that the LF thickness of the LFH specimens was > 3.74 mm (**Figure 6A2**). A comparison of clinical variables between the non-LFH group and LFH group is shown in **Table 3**.

**Table 3:** Data between the non-LFH and LFH groups.

Variable	Non-LFH Group (n = 8)	LFH Group (n = 8)	*P-*value
**Age (years)**	29.65 ± 3.79	63.50 ± 3.90	< 0.001
**Gender (male:female)**	5:3	4:4	–
**LF thickness (mm)**	2.57 ± 0.32	5.78 ± 0.60	< 0.001
**Lumbar level**	L4/5	L4/5	–

Independent sample t-test; data are presented as the mean ± SD; P < 0.05 is considered to be statistically significant. LF, ligamentum flavum; LFH, Ligamentum flavum hypertrophy.

Using TEM to observe the non-LFH and LFH specimens, it was found that cytoplasmic autophagy and mitochondrial autophagy existed in the non-LFH specimens (**Figures 6A3, A5**), while there were fewer autophagosomes in the LFH specimens (**Figures 6A4, A6**).

To further elucidate the relationship between LFH and autophagy, we examined the expression of the autophagy markers Beclin1 and P62 in the LF specimens of humans. Beclin1 is part of an autophagy-specific Class III phosphatidylinositol-3 kinase (PI3K) complex, which plays an important role in regulating autophagosome formation (30). P62 is one of the best-known autophagic substrates and, in contrast to Beclin1, its accumulation is observed when autophagy is inhibited (31). Results of IHC, qRT-PCR and Western Blotting revealed that Beclin1 was down-regulated in LFH specimens, whereas P62 was up-regulated (**Figures 6B–D**). In these findings, autophagy appears to play a protective role in LFH, as the level of autophagy declines with the progression of fibrosis.

### Verification of the mRNA expression of 14 hub ARGs in LFH patients

**Figure 7:**
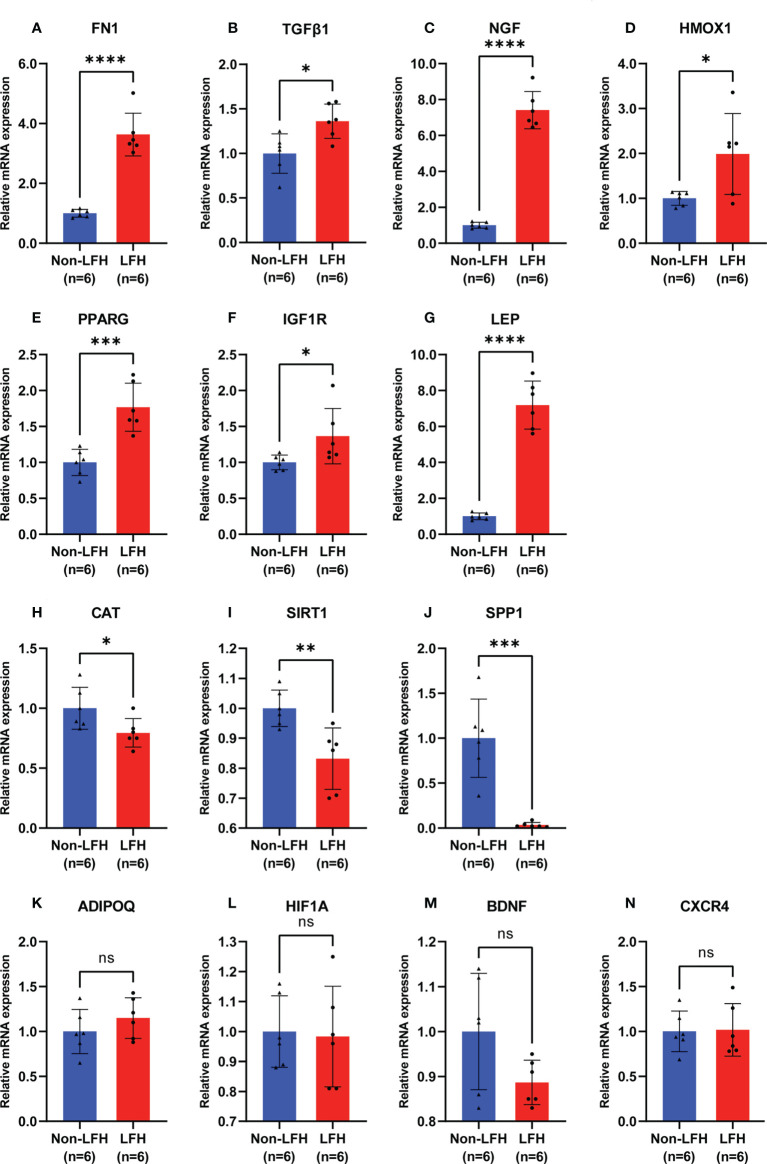
Validation of 14 hub ARG expression levels by qRT-PCR in specimens from the non-LFH and LFH groups. **(A–G)** The mRNA levels of *FN1, TGFβ1, NGF, HMOX1, PPARG, IGF1R, and LEP* were significantly higher in the LFH group than in the non-LFH group. **(H–J)** The mRNA levels of *SIRT1*, *CAT*, and *SPP1* were significantly decreased in the LFH group. **(K–N)** The mRNA levels of *ADIPOQ, HIF1A, BDNF, and CXCR4* were not significantly different between the non-LFH and LFH groups (non-LFH group:LFH group = 6:6). ns, not significant; *P < 0.05, **P < 0.01, ***P < 0.001, ****P < 0.0001.

To verify the accuracy of the bioinformatic analysis, we further identified 14 hub ARGs by using qRT-PCR to detect expression levels in clinical LF specimens. The relative mRNA levels of *FN1*, *TGFβ1*, *NGF*, *HMOX1, PPARG*, *IGF1R*, and *LEP* in LFH specimens were significantly higher than those in non-LFH specimens (**Figures 7A–G**). Compared with non-LFH patients, mRNA levels of *CAT*, *SIRT1*, and *SPP1* in LFH patients were significantly lower (**Figures 7H–J**). Among them, the mRNA levels of *FN1*, *TGFβ1*, *NGF*, *HMOX1*, *CAT*, and *SIRT1* were consistent with the mRNA microarray results, while *PPARG*, *IGF1R*, *LEP*, and *SPP1* had the exact opposite expression levels as the predicted microarray results. There were no significant differences between the two groups regarding expression levels of the mRNAs for *ADIPOQ*, *HIF1A*, *BDNF*, or *CXCR4* (**Figures 7K–N**).

### Verification of the protein expression of six hub ARGs in LFH patients

**Figure 8:**
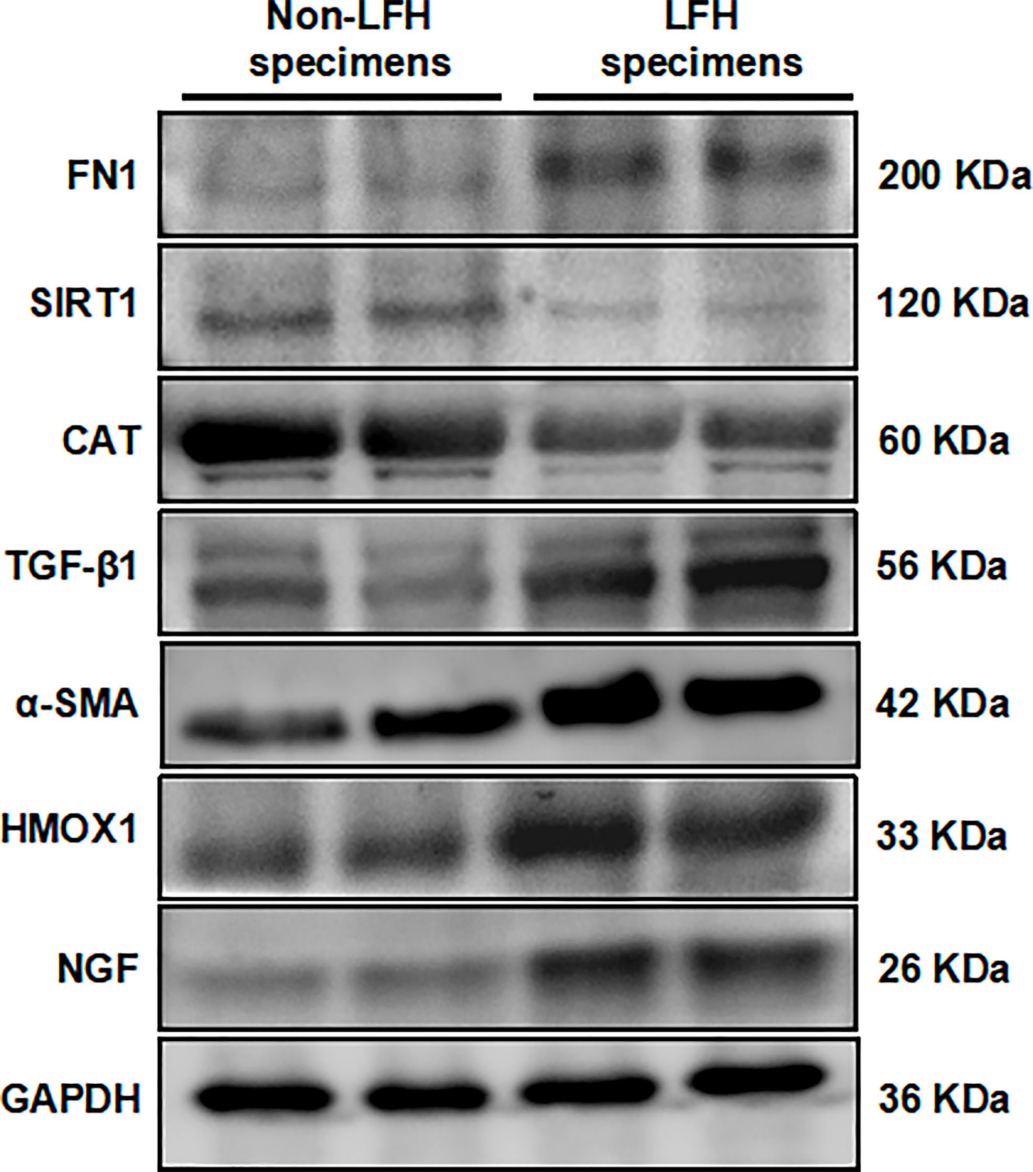
Validation of six hub ARG expression levels by Western blotting in specimens from the non-LFH and LFH groups. The protein expression levels of α-SMA, FN1, TGFβ1, NGF, and HMOX1 were upregulated in the LFH group compared with the non-LFH group. The mRNA levels *of SIRT1, CAT, and SPP1* were decreased in the LFH group (non-LFH group:LFH group = 2:2).

After studying the mRNA expression of 14 hub ARGs in LFH, we selected six ARGs whose results were consistent with that of the mRNA microarray results for further investigation. Western blotting results showed that FN1, TGFβ1, NGF, and HMOX1 protein levels were upregulated in LFH specimens compared with non-LFH specimens, while the protein levels of CAT, and SIRT1 were decreased (**Figure 8**). The expression of fibrosis marker α-SMA was upregulated in LFH specimens (**Figure 8**). The protein expression validations of FN1, TGFβ1, NGF, HMOX1, CAT and SIRT1 were completely consistent with our bioinformatics analysis.

### The verification of the expression of six hub ARGs in LFH patients and model mice

**Figure 9:**
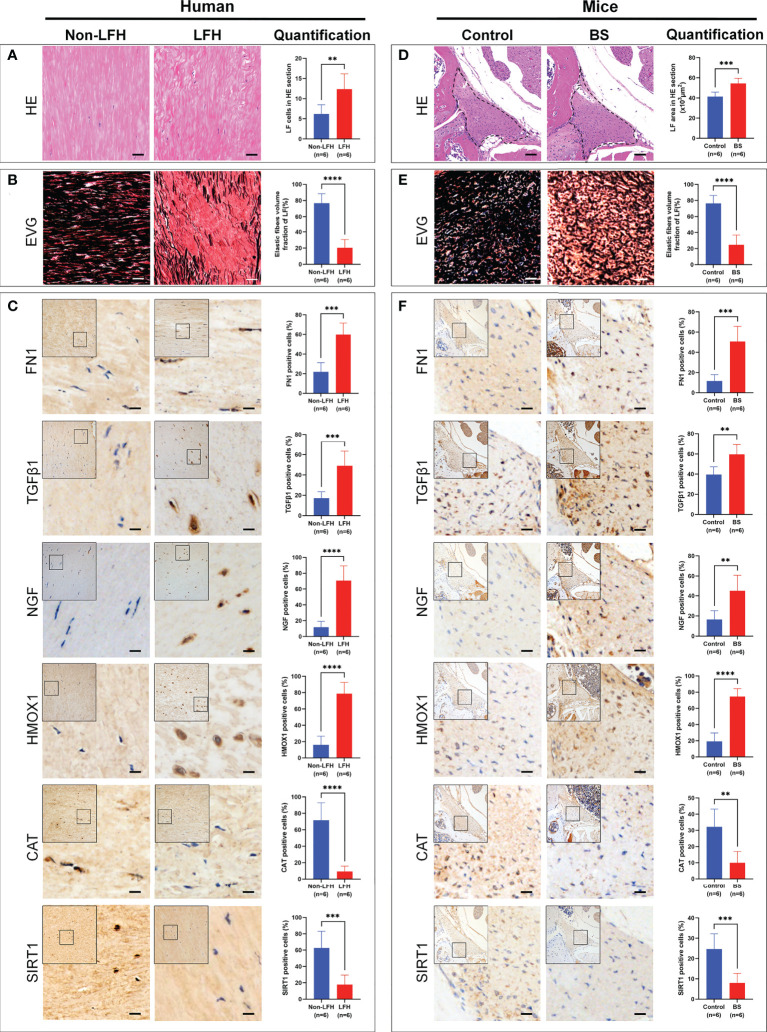
Validation of protein expression levels in LF specimens of patients and mice model. **(A)** HE staining of human LF specimens from the non-LFH and LFH groups. The bar chart shows quantitative analysis of LF cells in HE sections. **(B)** EVG staining of human LF specimens. Elastic fibers are dyed black and collagen fibers dyed red. The bar chart shows quantitative analysis of the elastic fibers volume fraction in human LF. **(C)** Representative IHC images of FN1, TGFβ1, NGF, HMOX1, CAT, and SIRT1 in the human specimens. Positive cells for each marker are stained brown, nuclei are stained blue. The bar charts show quantitative analysis of the ratio of positive cells of FN1, TGFβ1, NGF, HMOX1, CAT and SIRT1. **(D)** HE staining of mice LF specimens from control and BS groups. The bar chart shows quantitative analysis of the mouse LF tissue area in HE sections. **(E)** EVG staining of mouse LF specimens. Elastic fibers are dyed black and collagen fibers dyed red. The bar chart shows quantitative analysis of the elastic fibers volume fraction in mouse LF tissue. **(F)** Representative IHC images of FN1, TGFβ1, NGF, HMOX1, CAT, and SIRT1 in mouse specimens. Positive cells for each marker are stained brown, nuclei are stained blue. The bar charts show quantitative analysis of the ratio of positive cells of FN1, TGFβ1, NGF, HMOX1, CAT, and SIRT1. The scale is 50 μm. **P < 0.01, ***P < 0.001, ****P < 0.0001.

First, we confirmed that the LF specimens obtained from the LFH and BS groups exhibited greater degrees of fibrosis. HE staining showed that more LF cells could be observed in LF specimens from the LFH group than in the non-LFH group (**Figure 9A**), and the area of the LF in the BS group was larger than that in the control group (**Figure 9D**). Sections stained with EVG showed that collagen fibers increased and elastic fibers decreased significantly in the LFH group compared with the non-LFH group (**Figure 9B**), and the elastic fibers volume fraction of LF was lower in specimens of the BS group than in control specimens (**Figure 9E**).

Additionally, we measured the expression levels of Beclin1 and P62 in mouse LF specimens to determine whether the BS and control groups differ in autophagy. The IHC results demonstrated that, compared with the control group, the expression of Beclin1 of the BS group was lower in the mouse LF specimens, while the expression *of P62* was higher, which was consistent with findings in the control group (**Supplementary Figure 1**).

Next, we identified the expression level *of FN1, TGFβ1, NGF, HMOX1, CAT and SIRT1* in LFH patients and the BS model mice. IHC analysis showed that compared with the non-LFH group, the protein expression levels of FN1, TGFβ1, NGF, and HMOX1 in LF specimens of LFH patients was significantly increased, while CAT and SIRT1 were significantly decreased (**Figure 9C**). At the same time, compared with the control group, the protein expression levels of FN1, TGFβ1, NGF, and HMOX1 in LF specimens of the BS model mice also significantly increased, while CAT and SIRT1 significantly decreased (**Figure 9F**). These results showed that the expression validation was completely consistent with the results of our bioinformatics analysis.

## Discussion

As the most common reason for spinal surgery in patients over 65 years (2), LFH is widely considered a fibrotic disorder, which induces the accumulation of collagen fibers and ultimately leads to scar tissue formation. Despite autophagy being associated with fibrosis in different tissues, autophagy has not been reported to be involved in LFH. In this study, we first explored the relationship between autophagy and the fibrotic progression of LFH.

The process of tissue fibrosis is thought to be a form of abnormal wound healing that occurs after stimulation, in which the balance between collagen synthesis and degradation is disrupted (32). The pathological features of fibrogenesis in different tissues are similar, including inflammation, activation of myofibroblasts, and excessive ECM deposition (33). A series of recent studies has shown that autophagy plays a crucial role in the development of multi-organ fibrosis under various physiological and pathological conditions (16–18). Neither too little nor too much autophagy can be beneficial; however, uncontrolled autophagy may lead to cell death (34). Indeed, both enhanced and decreased autophagy have been associated with multi-organ fibrosis, highlighting the potentially diverse function of autophagy in the various phases of responses to stress and repair of damaged tissue (35). Thus, autophagy is involved in the regulation of fibrosis in a dual manner, and its clinical results can be affected by the tissue, cell type, and severity of the fibrosis.

In recent years, we have witnessed the continuous development of bioinformatics, which has accelerated the progress of research into the mechanisms of human disease. In the current study, we identified 70 potential ARGs in LFH through bioinformatics analysis. These differentially expressed ARGs were also analyzed by enrichment analysis for their potential biological functions. GO analysis of differentially expressed ARGs showed several enrichment terms related to autophagy and fibrosis, such as macroautophagy, mitochondrial autophagy, collagen metabolic process, and regulation of fibroblast proliferation. GSEA and GSVA results further revealed that ECM and collagen-related biological pathways were up-regulated in LFH, whereas autophagy-related biological pathways were down-regulated. Multiple signaling pathways obtained through KEGG analysis and GSVA have been proven to be related to autophagy, such as the PI3K-AKT, FoxO, AMPK, MAPK and mTOR signaling pathways (36). Furthermore, LFH has been reported to be associated with several KEGG pathways, such as the PI3K-AKT, MAPK, and mTOR signaling pathways (37–39).

TEM is currently the most conventional and straightforward technique for identifying autophagic structures (40). Using TEM, autophagy in LF specimens was further confirmed. In this study, autophagosomes and mitochondrial autophagy were first found in the LF at the ultrastructural level. We further evaluated the mRNA and protein levels of autophagy marker Beclin1 and autophagy substrate P62 in human specimens by qRT-PCR, Western blotting and IHC, respectively. We found that LFH specimens had fewer autophagosomes and that the mRNA and protein expression of *Beclin1* was reduced and that of *P62* was increased compared with non-LFH specimens, suggesting autophagy may play a protective role during the formation of LFH.

Previous studies have reported the pathological characteristics of human LFH samples as follows: elastic fiber fragmentation, collagen deposition, and increased expressions of fibrosis-related factors (6–10). To date, the molecular mechanisms of LFH have not been fully researched due to a lack of effective *in vivo* animal models. For medical research, mice are efficient and common animal models. Using mice models to study the pathological changes of LFH has received increasing attention in recent years, partly because they have the advantage of being affordable, accessible, and controllable in loading level (41, 42). In this study, we established the mouse LFH model as previously described based on the hydrophobic tendency of mice (29). This bipedal standing mouse model method has proven suitable for simulating the pathological process of LFH caused by mechanical stress in the human body (41). This bipedal standing mice have the advantage of providing a standardized condition for studying the molecular mechanisms underlying mechanical stress-induced LFH (29, 41). As a result of our study, the mouse LF specimens of the BS group had a greater level of fibrosis and an increased LF area, which is similar to the pathophysiology of LFH in humans. In addition, we found that Beclin1 expression levels of mRNA and protein decreased and P62 expression levels increased in LF specimens of mice after the 12-week bipedal modeling, which indicated that the autophagy level of the LF of mice in the BS group decreased during the formation of LFH under mechanical stress stimulation.

Based on the bioinformatics analysis results, qRT-PCR, Western blotting, and IHC were used to determine the expression levels of six differentially expressed ARGs in our clinical specimens and BS mice model. Experimental confirmation results indicated that the mRNA and protein expression levels of FN1, TGFβ1, NGF, HMOX1, CAT, and SIRT1 were consistent with our bioinformatics analysis, which verified the accuracy and reliability of bioinformatics analysis. To the best of our knowledge, the autophagy regulation mechanism of these six hub ARGs in fibrotic diseases has been studied. Evidence has indicated that autophagy inhibition may suppress the accumulation of *FN1* and apoptosis during the formation of kidney interstitial fibrosis (43). *TGFβ1* is a crucial molecular marker in fibrotic diseases, which has been shown to induce autophagy, apoptosis, and *FN1* accumulation in primary proximal tubular cells (43). *NGF* belongs to the NGFβ family, and has been found to have an profibrogenic effect on healthy-control primary cultures of conjunctival fibroblasts (44). *HMOX1*, one of the Heme oxygenase family’s main enzymes, plays a significant role in protection against oxidative injury and alleviation of cardiac fibrosis by regulating the transcription of key mitophagy proteins in Hmox1 knockout mice (45). *CAT* is one of the major intracellular antioxidant enzymes, which has been found to help alleviate autophagy in mice with cardiac injuries induced by diabetes (46). In addition, Odajima et al. found *CAT* to be protective against lung fibrosis (47). *SIRT1* is a nicotinamide adenosine dinucleotide-dependent deacetylase that inhibits cell apoptosis, inflammation, and fibrosis. It has been reported that cigarette smoke-inactivated SIRT1 promotes autophagy-dependent senescence of AT2 cells to induce pulmonary fibrosis (48). Besides *FN1*, *TGFβ1* has been shown to play an important role in LFH (42, 49), however, the mechanisms underlying the remainder of the hub ARGs (NGF, *HMOX1*, *CAT*, and *SIRT1*) in the occurrence and development of LFH have not been fully elucidated. Further study is necessary to discover their potential biological function.

Limitations to our study do exist. First, in our control group, we recruited young patients with lumbar disc herniation, as LF thickness is usually normal in young patients. Nonetheless, LFH is an age-related process (2). According to this classification, ARGs might be influenced by other aging processes. Second, studies on the mechanisms by which these six hub ARGs regulate LFH through autophagy are lacking *in vivo*. It will be necessary to further confirm the mechanism of these hub ARGs *in vivo*.

## Conclusions

In summary, we predicted 70 potential ARGs of LFH through bioinformatics analysis, and six hub ARGs of LFH were verified through experiments in clinical specimens and BS mice model. Our findings provide the evidences that autophagy may play a protective role in LFH, and the hub ARGs of *FN1*, *TGFβ1*, *NGF*, *HMOX1*, *CAT*, and *SIRT1* may be involved in the fibrosis development of LFH by regulating autophagy. These results help us better understand the pathological mechanism of LF fibrosis and provide the possibility for further study of the autophagy mechanism in LFH. We believe that the in-depth study of the autophagy regulation mechanism in LFH may provide new strategies for delaying or reversing fibrosis and provide new approaches and potential therapeutic targets for the treatment intervention of LFH patients.

## Data availability statement

The datasets presented in this study can be found in online repositories. The names of the repository/repositories and accession number(s) can be found in the article/**Supplementary Material**.

## Ethics statement

The studies involving human participants were reviewed and approved by ethics committee of Nanfang Hospital, Southern Medical University. The patients/participants provided their written informed consent to participate in this study. The animal study was reviewed and approved by Animal Ethical Committee of Laboratory Animals of Southern Medical University. Written informed consent was obtained from the individual(s) for the publication of any potentially identifiable images or data included in this article.

## Author contributions

LW and Z-MZ contributed to the study’s conception and design as correspondence authors. PL, C-SF and Y-LC contributed equally to this work as co-first authors. C-SF applied for the GEO dataset analysis and bioinformatics analysis. Y-LC and Z-SC carried out molecular biological experiments. Imaging data collection and clinical specimens were collected by XX, J-LD, and J-XZ. The animal experiments were accomplished by Z-ML and Y-PY. R-QT finished the histological staining. PL, C-SF and YLC completed the data analysis and drafted the manuscript. LW and Z-MZ revised and finalized the manuscript. All authors commented on previous versions of the manuscript. All authors contributed to the article and approved the submitted version.

## Funding

This work was supported by National Natural Science Foundation of China (82072520 and 81874013).

## Conflict of interest

The authors declare that the research was conducted in the absence of any commercial or financial relationships that could be construed as a potential conflict of interest.

## Publisher’s note

All claims expressed in this article are solely those of the authors and do not necessarily represent those of their affiliated organizations, or those of the publisher, the editors and the reviewers. Any product that may be evaluated in this article, or claim that may be made by its manufacturer, is not guaranteed or endorsed by the publisher.
