# Supplementary figures and images for: Revealing the novel autophagy-related genes for ligamentum flavum hypertrophy in patients and mice model
Source: Front Immunol. 2022 Oct 5;13:973799. doi: 10.3389/fimmu.2022.973799 (PMC9581255; doi:10.3389/fimmu.2022.973799)

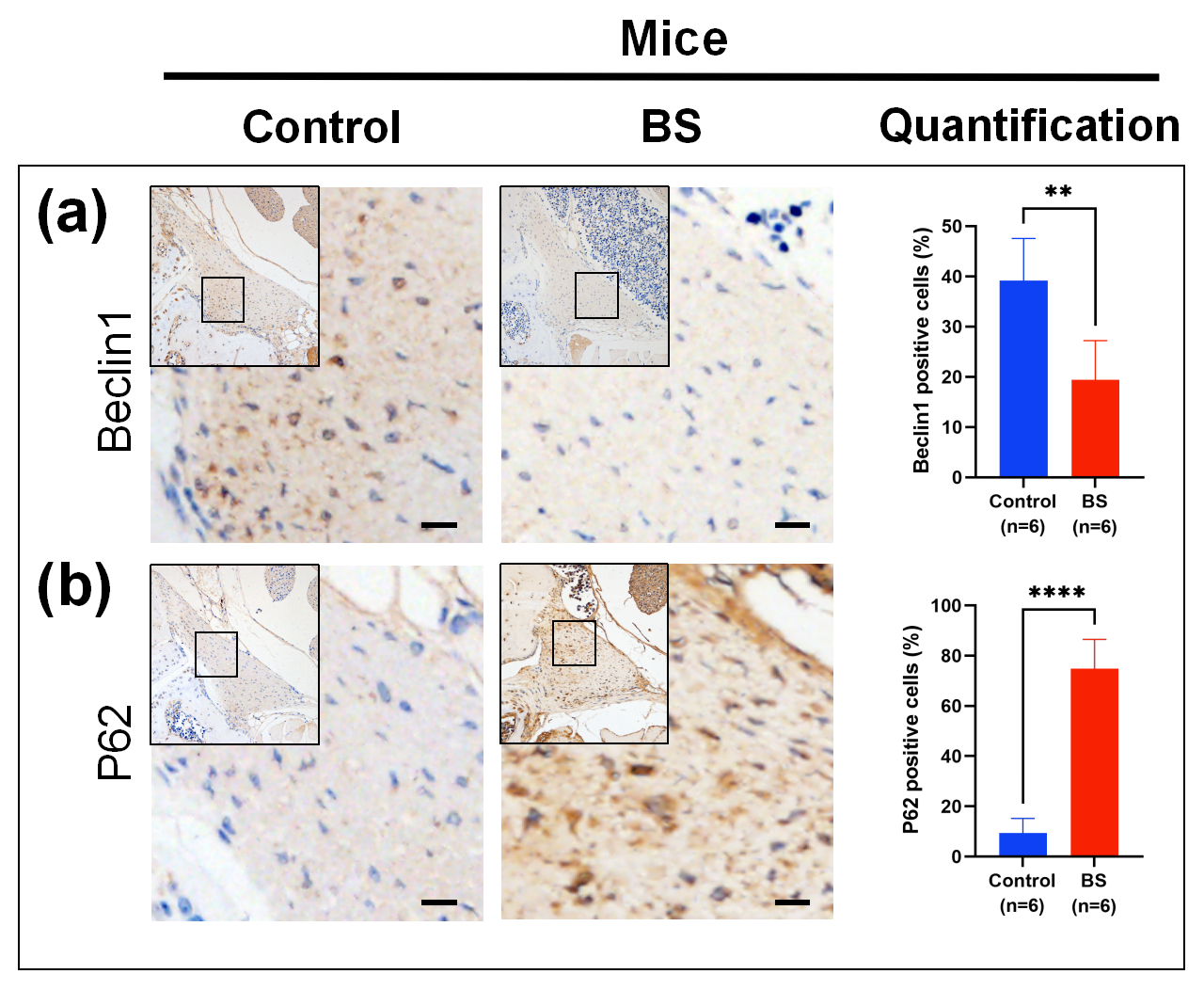

Supplement: Supplementary file 1 [file Image_1.tif]

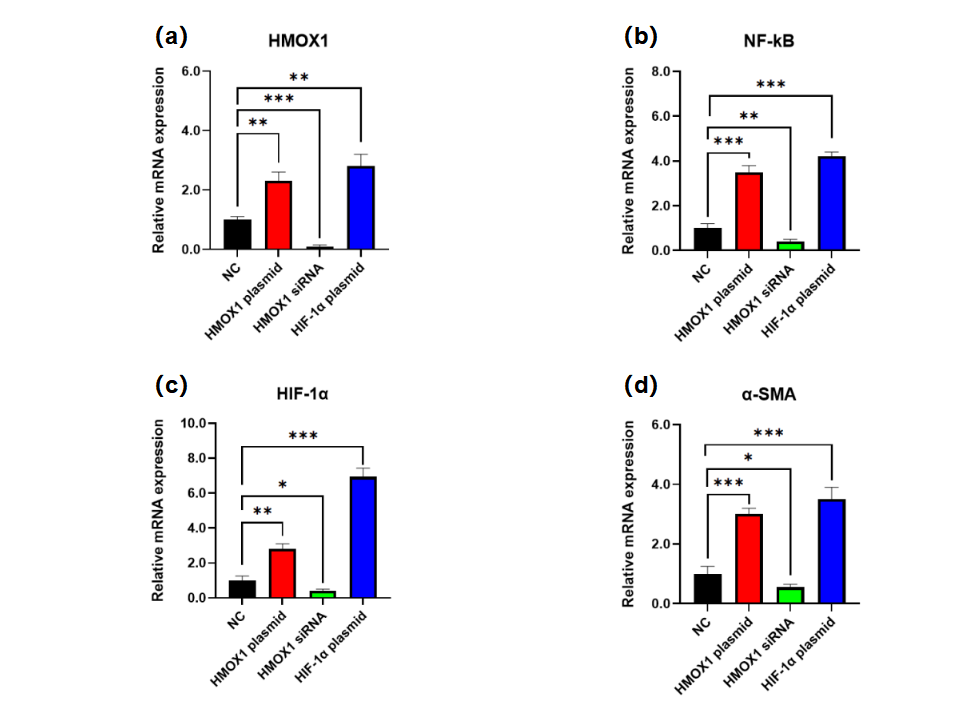

Supplement: Supplementary file 2 [file Image_2.tif]

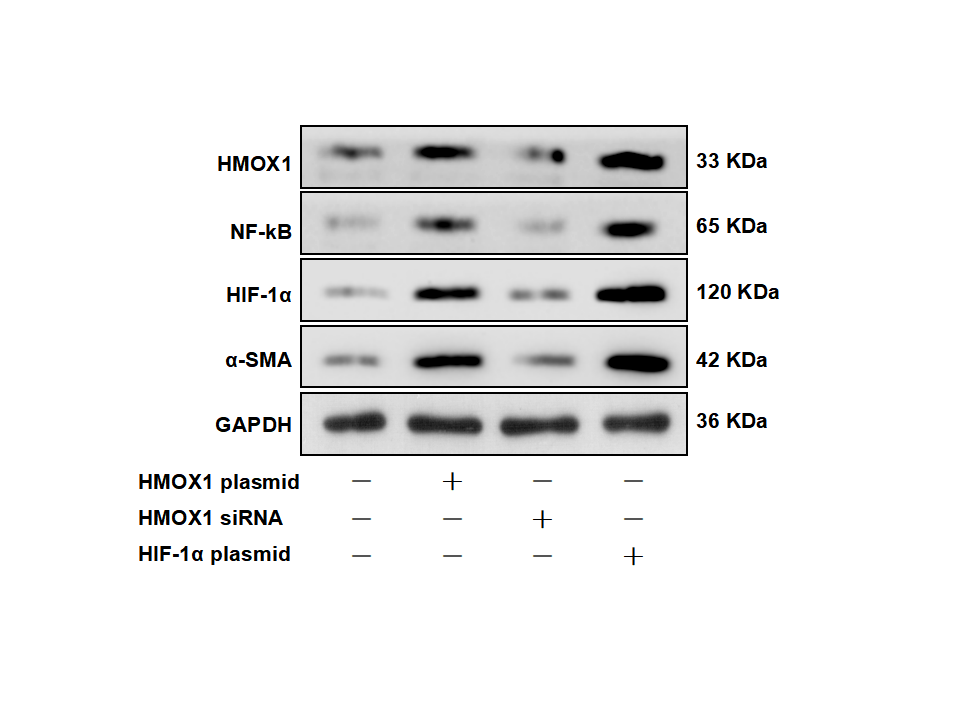

Supplement: Supplementary file 3 [file Image_3.tif]
