# Supplementary material for: Revealing the novel autophagy-related genes for ligamentum flavum hypertrophy in patients and mice model
Source: Front Immunol. 2022 Oct 5;13:973799. doi: 10.3389/fimmu.2022.973799 (PMC9581255; doi:10.3389/fimmu.2022.973799)
Supplement: Supplementary file 4 [file Table_1.docx]

**SUPPLEMENTARY TABLE 1** LF specimens for each experiment used in this study.

| **Total specimens** | **Storage** | **Experiments** |
| --- | --- | --- |
| 16 LF specimens (Non-LFH:LFH  = 8:8) were  equally cut into  32 LF specimens (Non-LFH:LFH  = 16:16) | 4 LF specimens were stored in 2.5% glutaraldehyde. | Transmission electron microscopy  (Non-LFH:LFH = 2:2) |
|  | 12 LF specimens were stored in PBS solution. | Histological staining assays  (HE, EVG, IHC)  (Non-LFH:LFH = 6:6) |
|  | 16 LF specimens were stored in liquid nitrogen.  (Non-LFH:LFH = 8:8) | Quantitative real-time PCR  (Non-LFH:LFH = 6:6) |
|  |  | Western blotting  (Non-LFH:LFH = 2:2) |
