# Supplementary material for: Revealing the novel autophagy-related genes for ligamentum flavum hypertrophy in patients and mice model
Source: Front Immunol. 2022 Oct 5;13:973799. doi: 10.3389/fimmu.2022.973799 (PMC9581255; doi:10.3389/fimmu.2022.973799)
Supplement: Supplementary file 5 [file Table_2.docx]

**SUPPLEMENTARY TABLE 2** The qRT- PCR primer sequences

| **Gene** | **Primer sequences** |
| --- | --- |
| ***SIRT1*** | F: 5′- ACTTCAGGTCAAGGGATGG - 3′  R: 5′- GTTCTGGGTATAGTTGCGAAG - 3′ |
| ***PPARG*** | F: 5′- GCCATCCGCATCTTTCAG - 3′  R: 5′- AGGCTTTCGCAGGCTCTT - 3′ |
| ***CAT*** | F: 5′- CCATTGCCACAGGAAAGTAC - 3′  R: 5′- ATGAGAGGGTAGTCCTTGTGA - 3′ |
| ***TGFβ1*** | F: 5′- CACGTGGAGCTGTACCAGAA - 3′  R: 5′- GAACCCGTTGATGTCCACTT - 3′ |
| ***IGF1R*** | F: 5′- TTTCCCTTTGGAGTGTAGCT - 3′  R: 5′- CATTGGCTGTGCAGTCAAG - 3′ |
| ***FN1*** | F: 5′- ATGATGAGGTGCACGTGTGT - 3′  R: 5′- CCCTGACCGAAGCATGTACA - 3′ |
| ***HMOX1*** | F: 5′- CCAGCAACAAAGTGCAAGAT - 3′  R: 5′- CATGGCATAAAGCCCTACAG - 3′ |
| ***LEP*** | F: 5′- CACCGGTTTGGACTTCATTC - 3′  R: 5′- GGTTCTCCAGGTCGTTGGAT - 3′ |
| ***NGF*** | F: 5′- CCTTCAACAGGACTCACAGG - 3′  R: 5′- GTCTTATCCCCAACCCACAC - 3′ |
| ***SPP1*** | F: 5′- GCAGACCTGACATCCAGTAC - 3′  R: 5′- TAATCTGGACTGCTTGTGGC - 3′ |
| ***ADIPOQ*** | F: 5′- TCAGCATTCAGTGTGGGATT - 3′  R: 5′- TTACCAGTGGAGCCATCATA - 3′ |
| ***BDNF*** | F: 5′- AGTGCAATCCCATGGGTTAC - 3′  R: 5′- TATGAATCGCCAGCCAATTC - 3′ |
| ***CXCR4*** | F: 5′- ATCAGTCTGGACCGCTACCT - 3′  R: 5′- GTCATCTGCCTCACTGACGT - 3′ |
| ***HIF1A*** | F: 5′- CCGCTGGAGACACAATCATAT - 3′  R: 5′- TGCAGGGTCAGCACTACTTC - 3′ |
| ***NFKB1*** | F: 5′- AACAGAGAGGATTTCGTTTCCG - 3′  R: 5′- TTTGACCTGAGGGTAAGACTTCT - 3′ |
| ***GAPDH*** | F: 5′- GGGAAACTGTGGCGTGAT - 3′  R: 5′- GAGTGGGTGTCGCTGTTGA - 3′ |
