# Supplementary material for: Revealing the novel autophagy-related genes for ligamentum flavum hypertrophy in patients and mice model
Source: Front Immunol. 2022 Oct 5;13:973799. doi: 10.3389/fimmu.2022.973799 (PMC9581255; doi:10.3389/fimmu.2022.973799)
Supplement: Supplementary file 6 [file Table_3.docx]

**SUPPLEMENTARY TABLE 3** The 70 Differentially Expressed ARGs in the LFH Samples Compared with the non-LFH Samples

| **Gene Symbol** | **LogFC** | **Changes** | ***P*-value** | **Chromosome** |
| --- | --- | --- | --- | --- |
| *IL11*  *FN1*  *SPP1*  *HMOX1*  *CTSD*  *SERPINA1*  *MCOLN3*  *ALPL*  *TNF*  *TLR7*  *NGF*  *RUNX1*  *CLEC11A*  *EPHB2*  *ENG*  *TGFB1*  *UBASH3B*  *KIF5C*  *ATG12*  *BCL2L11*  *CXCR4*  *SMPD1*  *AP1B1*  *LZTS1*  *MEGF10*  *ATG9A*  *RNF152*  *PRKCA*  *QSOX1*  *HSF1*  *ACP2*  *CTSB*  *HSPA5*  *SLC7A5*  *PSAP*  *CLTC*  *PPP2R5C*  *ERO1L*  *USP30*  *LEPR*  *SPNS1*  *RASD2*  *TAS1R3*  *ARSB*  *P4HB*  *ARSA*  *CHMP4B*  *GAA*  *CDKN1A*  *HIF1A*  *GRID2*  *SYNPO2*  *SIRT1*  *IGF1R*  *CAT*  *BDNF*  *MAPT*  *ACSL1*  *IL6*  *PPARG*  *MYOCD*  *LEP*  *KIF25*  *DIRAS3*  *SOX2*  *ADIPOQ*  *VPS18*  *EGFR*  *BCR*  *GPR182* | 3.7609375  3.6434375  2.90796875  2.86328125  2.18703125  2.12  2.0403125  1.97703125  1.97109375  1.913125  1.88046875  1.86859375  1.7159375  1.69625  1.65203125  1.6396875  1.63671875  1.633125  1.58671875  1.53390625  1.5284375  1.52828125  1.46265625  1.42625  1.3596875  1.3575  1.3496875  1.2921875  1.28  1.27453125  1.26  1.2484375  1.2453125  1.23140625  1.1434375  1.12390625  1.119375  1.118125  1.11625  1.10109375  1.09828125  1.07796875  1.0746875  1.0575  1.0421875  1.0334375  1.0153125  1.0109375  1.01  1.001875  −1.00484375  −1.01109375  −1.04640625  −1.05265625  −1.1053125  −1.19390625  −1.20375  −1.24375  −1.29625  −1.55875  −1.64515625  −1.66375  −1.716875  −1.7209375  −2.37234375  −2.391875  −2.9975  −3.65640625  −3.95921875  −5.13578125 | up  up  up  up  up  up  up  up  up  up  up  up  up  up  up  up  up  up  up  up  up  up  up  up  up  up  up  up  up  up  up  up  up  up  up  up  up  up  up  up  up  up  up  up  up  up  up  up  up  up  down  down  down  down  down  down  down  down  down  down  down  down  down  down  down  down  down  down  down  down | 0.000474949  0.001387795  0.014230078  0.010644373  0.000851453  0.001384223  0.013837538  0.018079495  0.015274992  0.007424524  0.006877428  0.008898808  0.040807868  0.01626061  0.000886956  1.56E-05  0.01966241  0.004142708  0.036221144  0.044111692  0.032981525  0.006735668  0.004245976  0.044839476  0.00931009  0.028240933  2.15E-05  0.00495358  0.00016416  0.008388359  0.010114851  0.008936885  0.012338302  0.044433769  0.000206593  0.011301697  0.00273546  0.002666055  0.034482366  0.041552047  0.006213882  0.036526391  0.002340593  0.003309584  0.001123761  0.008352786  0.008261955  0.000348773  0.003017797  0.010342145  0.048568169  0.016754498  0.010585262  0.004832529  0.002697511  0.007792565  0.004339201  0.017417131  0.036590543  0.001742071  0.025486129  0.034402676  0.048601385  0.000145866  0.003334328  0.002620062  0.004103073  0.000151684  0.000319759  0.000729553 | 19q13.42  2q35  4q22.1  22q12.3  11p15.5  14q32.13  1p22.3  1p36.12  6p21.33  Xp22.2  1p13.2  21q22.12  19q13.33  1p36.12  9q34.11  19q13.2  11q24.1  2q23.1-q23.2  5q22.3  2q13  2q22.1  11p15.4  22q12.2  8p21.3  5q23.2  2q35  18q21.33  17q24.2  1q25.2  8q24.3  11p11.2  8p23.1  9q33.3  16q24.2  10q22.1  17q23.1  14q32.31  14q22.1  12q24.11  1p31.3  16p11.2  22q12.3  1p36.33  5q14.1  17q25.3  22q13.33  20q11.22  17q25.3  6p21.2  14q23.2  4q22.1-q22.2  4q26  10q21.3  15q26.3  11p13  11p14.1  17q21.31  4q35.1  7p15.3  3p25.2  17p12  7q32.1  6q27  1p31.3  3q26.33  3q27.3  15q15.1  7p11.2  22q11.23  12q13.3 |
