# Supplementary material for: Revealing the novel autophagy-related genes for ligamentum flavum hypertrophy in patients and mice model
Source: Front Immunol. 2022 Oct 5;13:973799. doi: 10.3389/fimmu.2022.973799 (PMC9581255; doi:10.3389/fimmu.2022.973799)
Supplement: Supplementary file 7 [file Table_4.docx]

**SUPPLEMENTARY TABLE 4** Abbreviations List

| **Full name** | **Abbreviations** |
| --- | --- |
| Ligamentum flavum hypertrophy | LFH |
| Ligamentum flavum | LF |
| Degenerative lumbar spinal stenosis | DLSS |
| Lumbar disc herniation | LDH |
| Extracellular matrix | ECM |
| Differentially expressed genes | DEGs |
| Autophagy-related genes | ARGs |
| Gene Expression Omnibus database | GEO |
| Molecular Signatures Database | MSigDB |
| Gene Ontology | GO |
| Kyoto Encyclopedia of Genes and Genomes | KEGG |
| Biological process | BP |
| Cellular component | CC |
| Molecular function | MF |
| Gene Set Enrichment Analysis | GSEA |
| Gene Set Variation Analysis | GSVA |
| Protein-Protein Interaction | PPI |
| Maximal clique centrality | MCC |
| Density of maximum neighborhood component | DMNC |
| Maximum neighborhood component | MNC |
| Magnetic resonance imaging | MRI |
| Transmission Electron Microscopy | TEM |
| Quantitative RT-PCR | qRT-PCR |
| Bipedal standing | BS |
